# Supplementary figures and images for: Ablation of non-coding RNAs affects bovine leukemia virus B lymphocyte proliferation and abrogates oncogenesis
Source: PLoS Pathog. 2020 May 14;16(5):e1008502. doi: 10.1371/journal.ppat.1008502 (PMC7252678; doi:10.1371/journal.ppat.1008502)

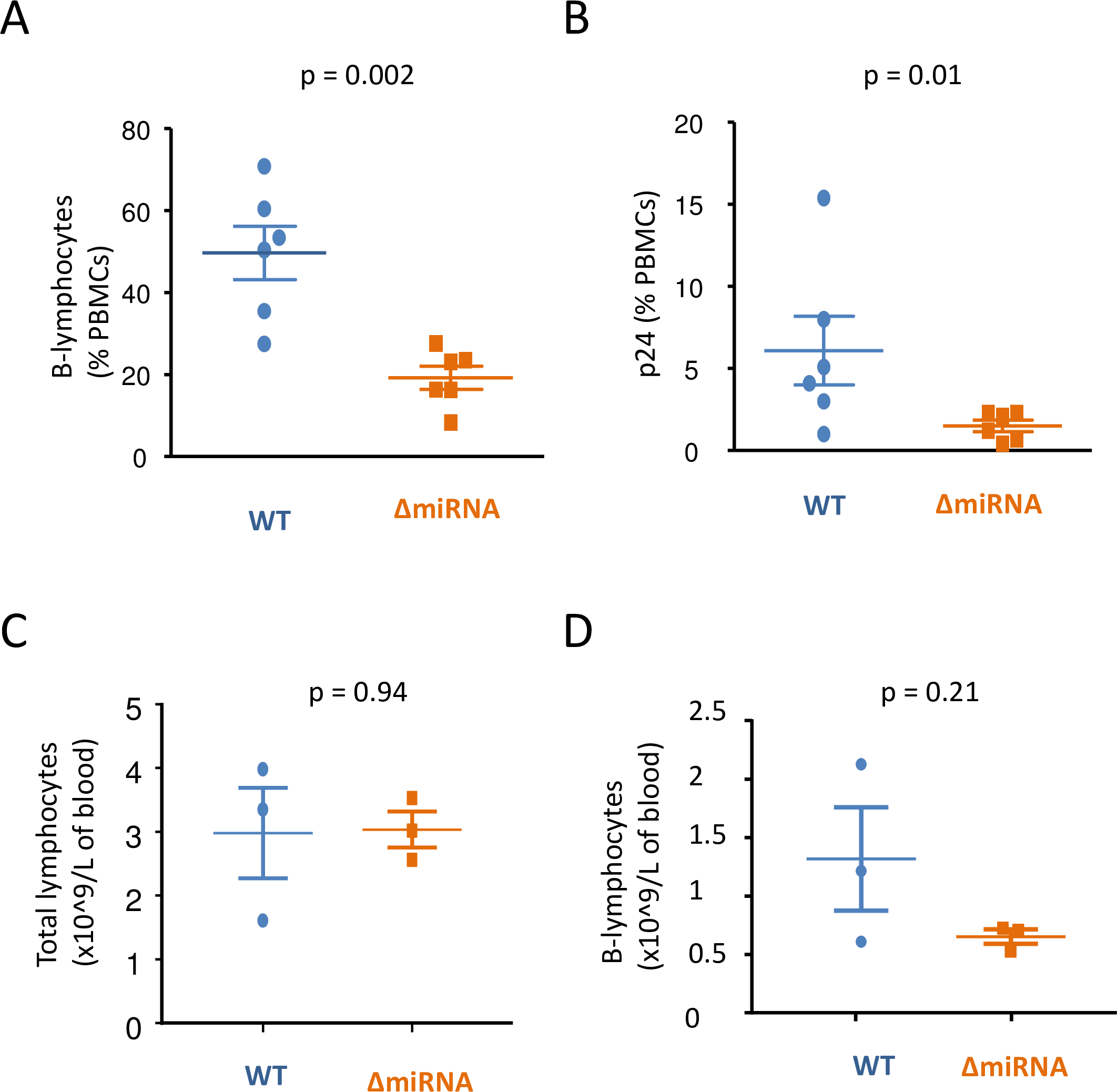

Supplement: S1 Fig — (A) PBMCs were isolated from sheep infected by wild-type BLV and miRNA deletant. Then, PBMCs were labeled with anti-IgM 1H4 antibody and anti-mouse IgG1 conjugate. Percentages of B cells were determined by flow cytometry. p = 0.002 according to Mann-Whitney U test. (B) After overnight culture of PBMCs, the percentages of p24-positive cells were determined by flow cytometry. The p24 viral protein was detected by sequential incubation with 4′G9 monoclonal antibody and a rat anti-mouse IgG1 conjugate. p = 0.01 according to Mann-Whitney U test. (C) Absolute number of lymphocytes determined with a ProCyte Dx Haematology Analyser. (D) Absolute numbers of B cells were calculated from the percentages of B-lymphocytes in PBMCs (panel A) and absolute numbers of lymphocytes (panel C). (TIF) [file ppat.1008502.s001.tif]

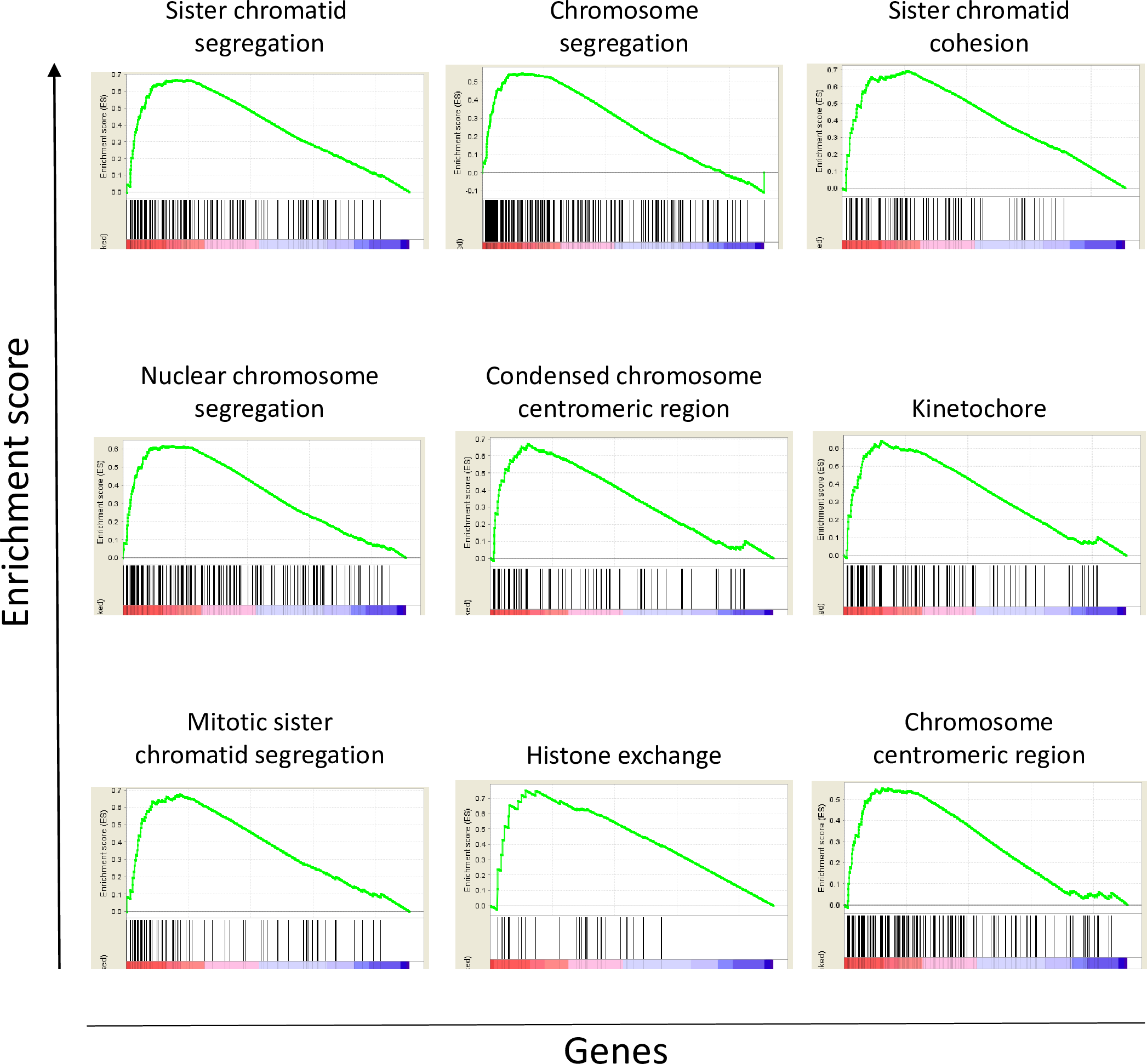

Supplement: S2 Fig — The green curve corresponds to the ES (enrichment score) curve, which is the running sum of the weighted enrichment score obtained from GSEA software. The enrichment score reveals the degree at which the genes in a gene set are overrepresented at the top or bottom of the entire ranked list of genes (y axis). (TIF) [file ppat.1008502.s002.tif]

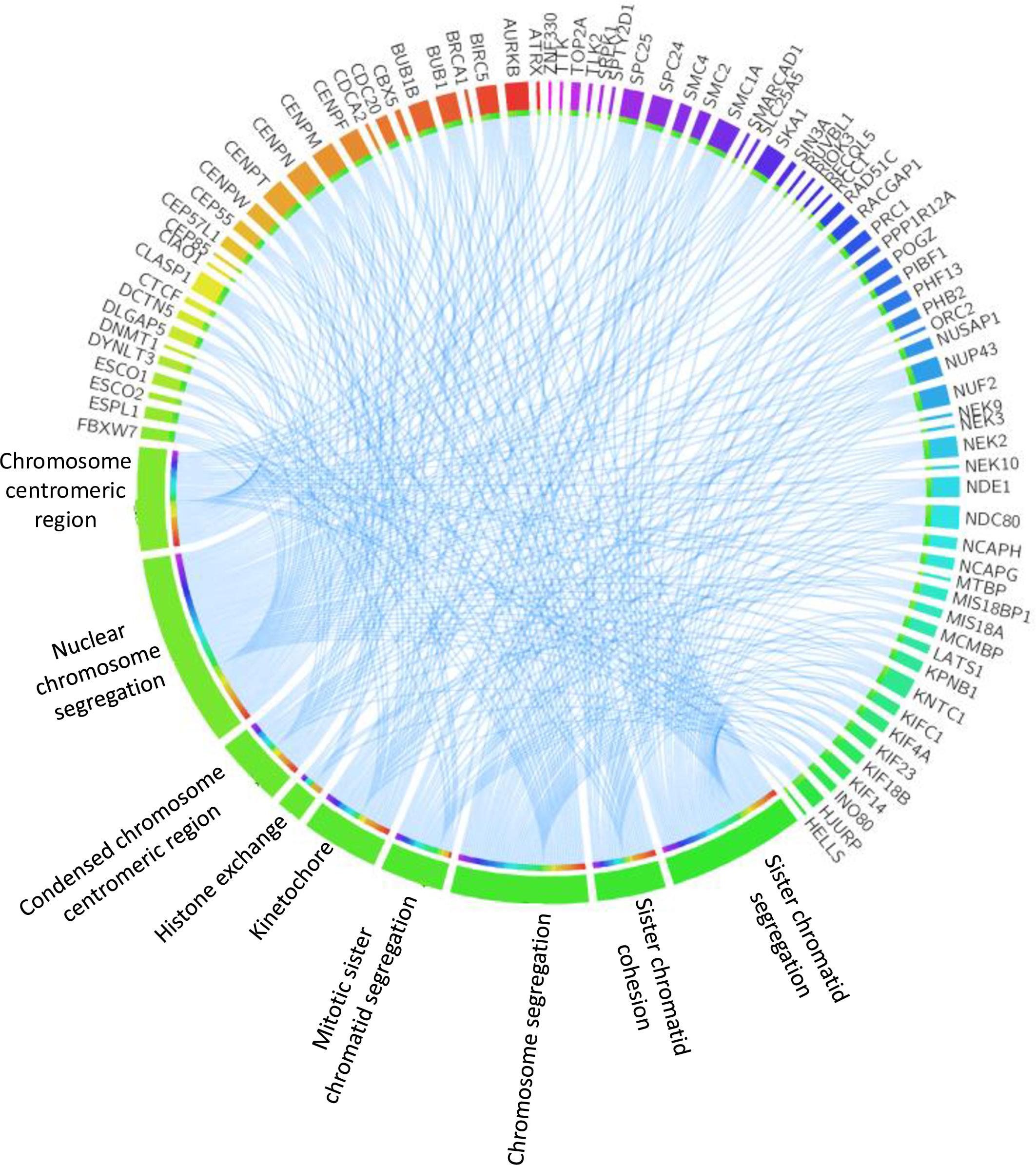

Supplement: S3 Fig — Chord diagram displaying leading edge analysis of enriched gene sets (FWER < 0.001) in pBLV-WT-infected sheep analyzed by GSEA. The diagram was generated by circos table viewer. Segments size shows the contribution effect. (TIF) [file ppat.1008502.s003.tif]

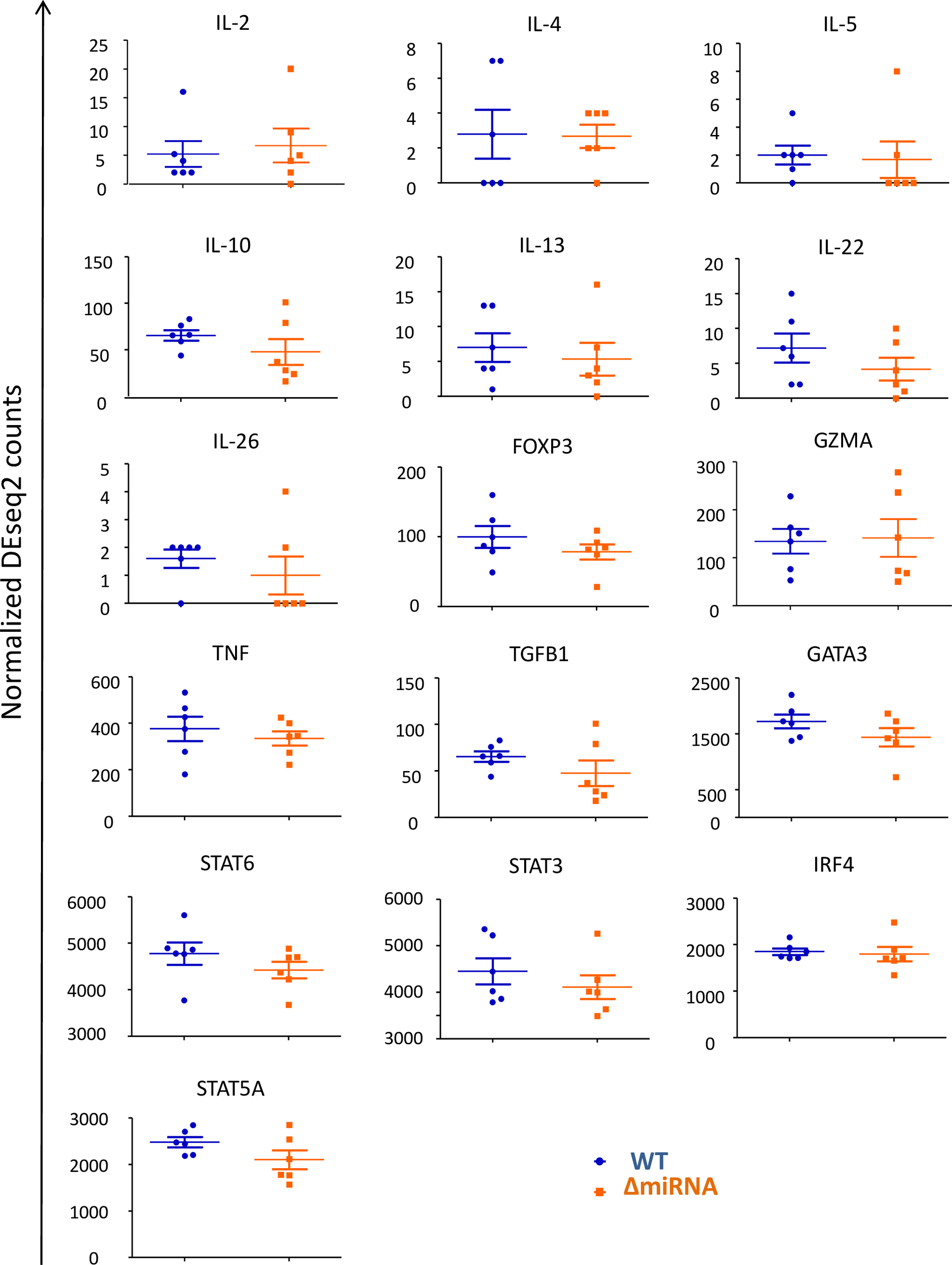

Supplement: S4 Fig — Normalized counts were obtained by DEseq2 analysis of transcriptomic data of non-B cells isolated from pBLV-WT and pBLV-ΔmiRNA infected sheep. Differences of gene expression between pBLV-WT and pBLV-ΔmiRNA are not significant according to t-test. (TIF) [file ppat.1008502.s004.tif]

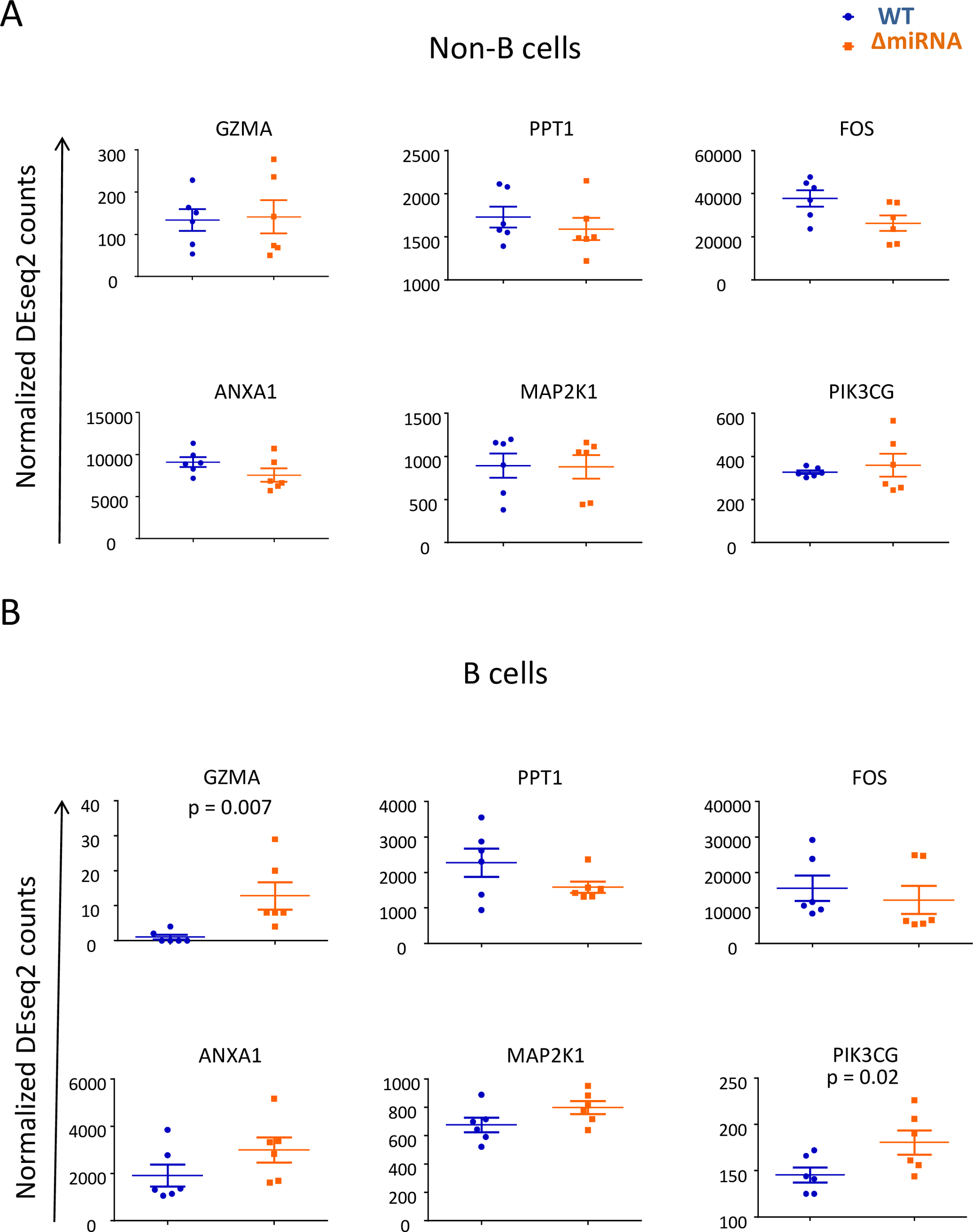

Supplement: S5 Fig — (A) Normalized counts obtained from DEseq2 analysis of transcriptomic data of non-B cells isolated from pBLV-WT and pBLV-ΔmiRNA infected sheep. Differences of gene expression between pBLV-WT and pBLV-ΔmiRNA are not significant according to t-test. (B) Normalized counts obtained from DEseq2 analysis of B cells. Differences are significant for GZMA (p = 0.007) and PIK3CG (p = 0.02) according to t-test. (TIF) [file ppat.1008502.s005.tif]

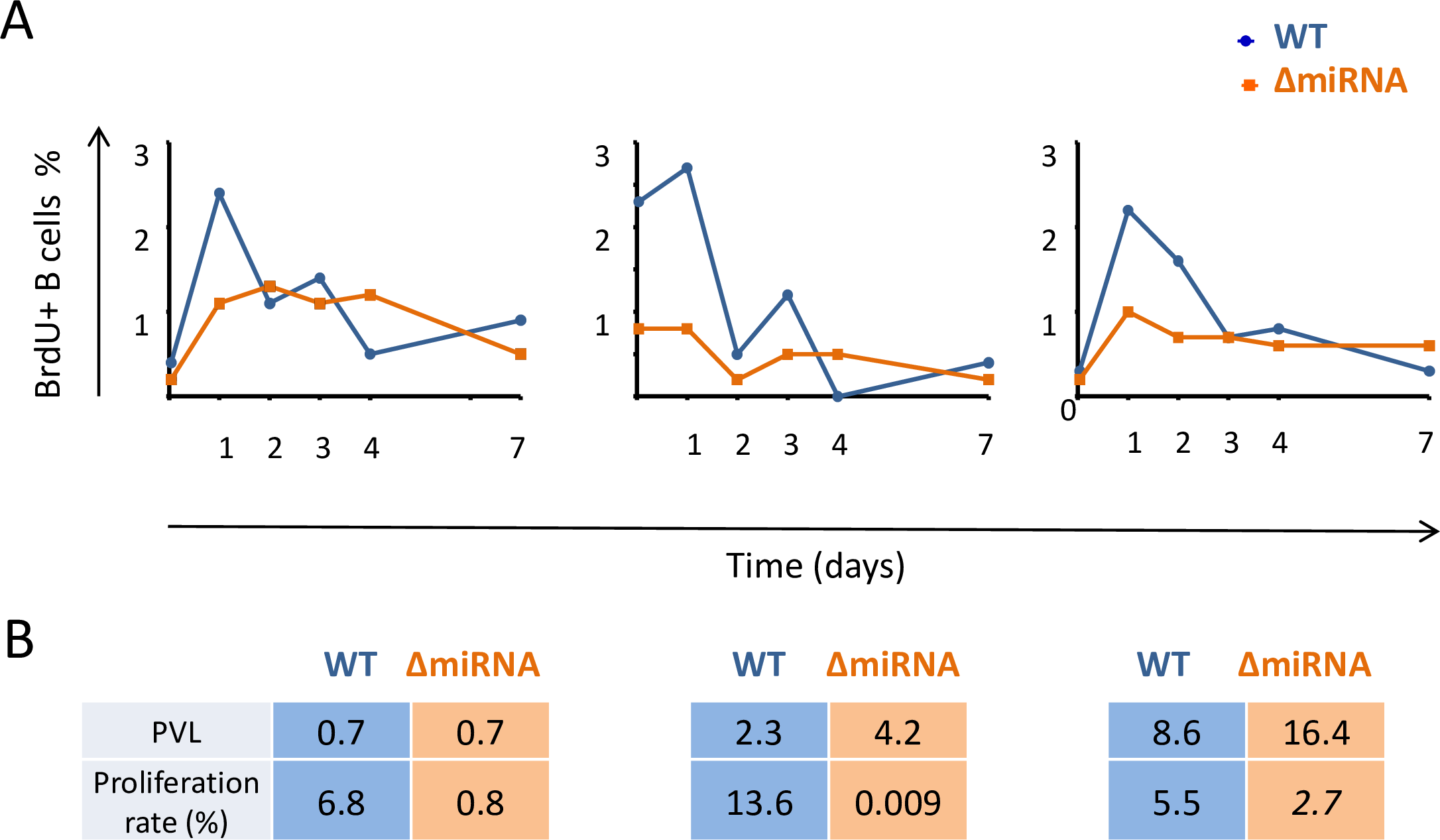

Supplement: S6 Fig — (A) Time kinetics of the percentages of B cells having incorporated BrdU. (B) Proviral loads (in number of copies in 100 PBMCs) and proliferation rates corresponding to graphs of panel A. (TIF) [file ppat.1008502.s006.tif]

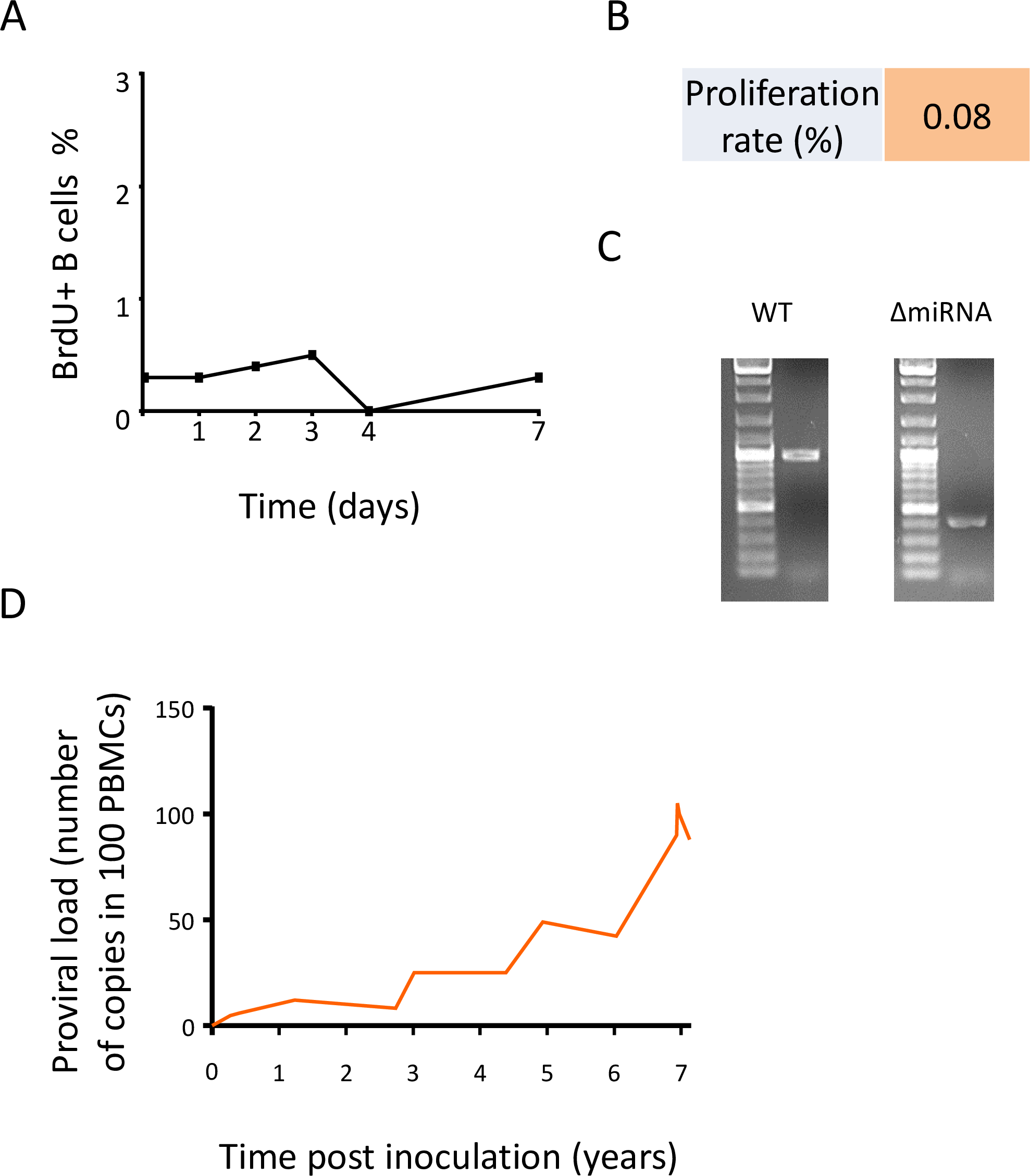

Supplement: S7 Fig — (A) Time kinetics of the percentages of B cells having incorporated BrdU in animal # 1131 infected with pBLV-ΔmiRNA (B) Proliferation rate estimated from data of panel A. (C) PCR amplification of the genomic sequences surrounding the miRNA region. (D) Kinetics of proviral loads (in number of copies in 100 PBMCs) in sheep #1131. (TIF) [file ppat.1008502.s007.tif]
