# Supplementary material for: Ablation of non-coding RNAs affects bovine leukemia virus B lymphocyte proliferation and abrogates oncogenesis
Source: PLoS Pathog. 2020 May 14;16(5):e1008502. doi: 10.1371/journal.ppat.1008502 (PMC7252678; doi:10.1371/journal.ppat.1008502)
Supplement: Attachment — Submitted filename: Response to reviewers.docx [file ppat.1008502.s012.docx]

We thank the reviewers for their appropriate and useful comments that significantly improved our manuscript. Please find below the specific answers to the requests.

**Part I - Summary**

**Reviewer #1:** It has been recognized for some years that bovine leukemia virus (BLV) encodes several RNAs that do not encode proteins, but the functions of these non-coding RNAs are not completely understood. In this paper, the authors use the well-established model of infection of sheep with BLV, to compare the wild type virus with the same strain from which the non-coding RNAs have been knocked out. They conclude that an important function of the non-coding RNAs is to promote proliferation of BLV-infected B cells in vivo. The paper is well written, the data are clearly presented and for the most part support the major conclusions drawn by the authors.

**Response:** We appreciate this positive comment.

**Reviewer #2:** Safari et al describe the role of BLV non-coding RNAs on viral replication, leukemia development and regulation of host cell gene expression in sheep using a reverse genetic approach. The same group has previously performed a very similar study (published in 2016) in a bovine model showing that the viral miRNAs regulate the expression of genes involved in cell signaling, cancer and immunity. As the approach used in the two studies is the same, the novelty of the data described in the current manuscript is limited. Although the sheep model allows to evaluate the long-term effects of the viral miRNAs compared to the bovine animal model, no mechanistic analyses have been included, thus limiting the impact of the new data compared to previous observations.

**Response:** The main focus of the paper is to provide information on cell dynamics in the absence of BLV miRNAs. In fact, we were surprised that the transcriptome in T cells was quite similar in sheep infected with wild-type and mutant viruses. As requested, the new version of the manuscript contains a comparison of the mechanisms occurring in cows and in sheep. In particular, we added an analysis of the relationship between proliferation, cell signaling, cancer and immunity (page 12). We also added information about the mechanisms that direct increased proliferation in particular the process of chromosome segregation at mitosis.

**Reviewer #3:** In this manuscript Safari et al described the role of non-coding RNA in the B-cell biology after experimental infection of sheep animals with BLV. They confirmed their previous observation, showing that sheep infected with BLV devoided of miRNA have a reduced viral load compare to infection with WT virus and survive to the infection while sheep infected with virus didn't. In this study, the deeply analyzed the role of miRNA in B cell compartment. Using transcriptomic analysis of B-cells purified from PBMCs of sheep infected with miRNA deletant virus or WT virus, they identified that viral miRNA mainly target B-cell and not non-B cells population and mainly genes involved in proliferation pathways. Using, in vivo labeling they further demonstrated that B-cells from WT infected animals had a higher proliferation rate than B cells from miRNA deletant infected animals. The use of in vivo infection and analysis of freshly purified cells is certainly an important strength of this study.

**Response:** We appreciate this positive comment.

The main weaknesses are linked to the lack of mechanisms supporting the lower B-cell proliferation rate after infection with miRNA deletant. Specially, since the author have reported a role of miRNA in controlling expression of gene involved in cell signaling, cancer and immunity, it would have been interested to address whether these gene are involved in B-cell proliferation and or also upregulated in B cell from animals infected with miRNA deletant.

**Response:** As requested, we have now added an analysis of the relationship between proliferation, cell signaling, cancer and immunity (page 12). We also provide data on upregulated pathways in B cells of pBLV-ΔmiRNA infected sheep as compared to pBLV-WT (Supplementary Table 4).

In addition, it would be interested to determine whether the 2x increase in B cells proliferation observed in WT infected animals translated to higher B cells total count reminiscent to oncogenesis.

**Response:** We have added data (Figure S1 panels C and D) showing that the sheep infected with wild-type and mutant virus have similar B cell counts. The absolute B cell number is not statistically significantly different (p=0.21) but the cell turnover (including proliferation and death) is reduced in the absence of miRNAs.

**Part II – Major Issues**

**Reviewer #1:**

**1.1.** Throughout the paper, the emphasis presented by the authors is on the impact on proliferation of knocking out the non-coding RNAs. However, the gene ontology analysis (results summarized in Figure 3) appears to suggest a more specific effect than merely on proliferation. Most of the genes whose expression is altered in the B cell infected by the knockout virus are concerned with the mechanics of chromosome segregation at mitosis. Obviously, these changes will be associated with a change in the cell proliferation rate. But these results suggest that the microRNAs might be exerting an impact on one particular stage of mitosis, and might even suggest some candidate targets of the non-coding RNAs. The authors might comment on this, if they agree, in the discussion. It would be very interesting to pursue this in future work.

**Response:** As requested, we have added a paragraph in the discussion about the possible mechanisms that may direct increased proliferation in particular the process of chromosome segregation at mitosis. The gene ontology analysis (Figure 3) in B cells indeed indicates a more specific effect of the miRNAs on mitosis than merely on proliferation. Most genes whose expression is increased in the presence of miRNAs concern chromosome segregation at mitosis (Figure S3 and Supplementary Tables 2 and 3). These include AURKB, the serine/threonine-protein kinase component of the chromosomal passenger complex (CPC), a complex that acts as a key regulator of mitosis. This is now added these data in the text (pages 12 and 13).

**1.2**. Lines 254-261. It is a curious observation that the B-cell proliferation rate differed between the two groups of animals even when the proviral loads were very similar. This observation also implies that the risk of disease (leukemia), which differed between the groups (cf. survival curves, Fig. 1B), might depend on the viral genotype independently of the proviral load. In turn this raises the question whether there is a difference between the groups in the clonal composition of what is presumably a highly polyclonal infected B-cell population, i.e. whether there is selection for particular different BLV-infected B-cell clones, which differ in leukemogenic potential. Have the authors studied the clonality of these populations?

**Response:** The fact that the B-cell proliferation rates differ between the two groups of animals even when the proviral loads are similar is indeed an unexpected conclusion of our study. It is possible that the risk of disease might depend on the viral or host genotypes independently of the proviral load. As indicated, there might be a difference in the clonal evolution of the infected cells. Alternatively, the miRNAs that are known to be exported in the plasma may affect proliferation of non-infected B cells. In fact, this mechanism may also explain the increase of infected as well as non-infected B-cells during persistent lymphocytosis (PL) in cows. We have now discussed this point in page 14.

**1.3.** Line 175 and fig. 4C. It is incorrect to use the t-test (whether paired or not) to test for a possible difference between the two curves in Fig. 4C. ... Similarly, one cannot legitimately use regression techniques to test for a difference in the location of the two curves, again because of the non-independence of the plotted points.

**Response:** As also requested by reviewer 2, we have corrected the statistical analyses by using non-linear mixed models. Accordingly, we revised the analysis, interpretation and conclusions in the manuscript.

**Reviewer #2:**

**2.1.** Authors have identified cell division as the major pathway regulated by BLV miRNAs in infected sheep. However, authors do not provide a detailed analysis of potential key genes in this pathway which could be directly affected by miRNAs and that could explain the different phenotype caused by the WT virus and the ΔmiRNA virus.

**Response:** As requested, we have now provided a detailed analysis of key genes involved in major pathways regulated by BLV miRNAs (Supplementary Table 2). The major genes altered by BLV miRNAs concern the mechanics of chromosome segregation at mitosis. See also response 1 to reviewer 1. This is now indicated in the discussion in page 12 and 13.

**2.2.** What other pathways, besides cell division, are affected by the absence/presence of viral miRNAs? Authors mention in the discussion that in bovine PBMCs the miRNAs modulated a more complex network of pathways, but they do not discuss a more detailed comparison of the results observed in the two animal models, which would instead be interesting, e.g. is the gene expression profile in bovine and ovine cells very different? Are the pathways identified in the bovine model (cell signaling, cancer and immunity) also affected in sheep?

**Response:** Besides cell division, the most affected pathways by the presence of BLV miRNAs pertain to DNA repair (Supplementary Tables 2 and 3). The most affected pathways correlating with absence of BLV miRNAs include inflammation response, immunity and cell signaling (Supplementary Table 4). The pathways identified in the bovine model (cell signaling, cancer and immunity) are thus also affected in sheep. For clarification, we added 3 tables showing the pathways that are enriched by the absence/presence of BLV miRNAs. We have modified the manuscript accordingly and included a more detailed comparison of the results observed in the two animal models (page 12).

**2.3.** Fig 4: why the 2-fold increase shown in panel A at 1 month is not reflected in panel C?

**Response:** Panel A is showing the data for only one animal in each group. Panel C is representing the mean data ± SEM. Means ± SEM are 15.56 ± 3.92 and 17.8 ± 3.4 in the wild-type and mutant groups, respectively. We added this information in the figure legend.

**2.4.** In panels C and D, authors state that viral miRNAs significantly control B cell turnover, cell proliferation and death, but the percentage of CFSE+ cells, proliferation rate and death rate are very similar between WT and ΔmiRNA viruses, with a fold difference less than 2. It’s not clear how biologically relevant this difference is. The authors should comment on this point?

**Response:** As indicated in the response to referee 1, it is correct that the CFSE profiles are similar based on non-linear mixed models. Since the value at day 23 is statistically different (Figure 4C), there is nevertheless a trend to a faster decrease in the presence of miRNAs that may be biologically relevant. The two-fold increase in BrdU incorporation that occurs in lymphoid organs should be compensated by a higher death rate in the peripheral blood. We have previously published a model that reconciles these apparent discrepancies (see reference #36). This is now indicated in page 13.

**2.5.** Fig 5A:

- At what point of the study was the BrdU analysis done?

- In the material and methods section, authors state that PBMCs were isolated at different time points after BrdU injection. It would be interesting to see the BrdU incorporation data at later time points (day 2-17): does the difference between WT and ΔmiRNA viruses become more pronounced?

**Response:** We performed a single injection of BrdU at day 0 and analyzed data at different times (1 hour and days 1, 2, 3, 4, 7, 17). Mathematical modeling of the proliferation and death rates is possible using the percentages of BrdU+ B cells. When multiple injections of BrdU are performed, the total number of BrdU+ cells increases stepwise (Debacq and Florins, unpublished data). This indicates that the pool of proliferating cells is regularly renewed.

**Reviewer #3:**

**3.1.** Using the transcriptomic data set what is the B-cell and non-B cell expression of ANXA1, FOS, GZMA, PIK3CG and PPT1 that were previously shown to be controlled in PBMCs by BLV miRNA? And what is the link of these genes with B cell proliferation?

**Response:** As requested, we analyzed the expression levels of the genes that were previously identified as being controlled by the miRNAs in the bovine model. Among these, GZMA and PIK3CG are significantly downregulated in the presence of BLV miRNAs in the ovine model. We have added this information in Figure S6 and modified the discussion (page 12).

**3.2.** Transcriptomic pinpoint to B-cell proliferation that was confirmed by CSFE and BrdU labelling. However, the total count of B-cell was not provided in these experiments. What is the total number of B-cell in animals infected with miRNA deletant compare to animals infected with WT? Does this number correlate with the 2X increase in proliferation that was demonstrated in fig 4 and 5?

**Response:** We have included the numbers of total lymphocytes and the numbers of B-cells in blood (Figure S1, panels C and D). The 2X increase in proliferation is not reflected by the total number of cells. The data show that the cell turnover including proliferation and death is accelerated in the presence of miRNAs. See also answer to comment 2.4. of reviewer 2.

Is B-cell proliferation also observed after in vitro culture of purified B-cells? Are in vitro infected B-cells with miRNA deletant also impaired in their proliferation ability compared to WT in vitro infected B-cells?

**Response:** The available cell culture models are inappropriate to analyze B cell proliferation. In fact, B cell undergo massive spontaneous apoptosis but do not proliferate.

**3.3.** Does the B cell proliferation correlate with the PVL? Especially B-cell proliferation from animals with similar low PVL after infection with miRNA deletant or WT virus should be shown (p 12 lane 254-255)

**Response:** There is no correlation between B cell proliferation and PVL. As requested, we added proliferation rates from WT or mutant infected animals with similar PVL (Figure S5).

**3.4.** Are the B cells that proliferate infected? Since PVL and infection are lower in miRNA deletant infected animals, one could expect that lower infected cells would translate in lower proliferation rate as measured in bulk B-cell population.

**Response:** It is currently not possible to address this question directly in vivo. Despite our efforts, a double labeling of BrdU and proviral DNA is associated with excessive background. Ex vivo cell culture that would stimulate viral expression followed by flow cytometry is also inappropriate because of excessive B cell death. As indicated in the previous comment 3.3., there is no correlation between B cell proliferation and PVL (Figure S5). The miRNAs are thus associated with the turnover rates but are not the only determinants that determine the proviral loads.

**Part III – Minor Issues**

**Reviewer #1:**

- Figure 1. Can the authors comment on the discrepant results in the WT animal with the slow rise in PVL?

**Response:** We added a comment on a possible involvement of the genotype of the animal that has a low rise in PVL (page 14).

- line 123. The authors write that ‘There was a clear segregation of the two principal components (PC1 and PC2)...’ But in Figure 2A, PC2 appears to differ between WT and ΔmiRNA animals, whereas PC1 does not.

**Response:** We meant that, in B cells, there is segregation between WT and ΔmiRNA data based on PC1. PC1 also segregates B and non-B cells. We have corrected the text accordingly.

- line 131. Please expand the abbreviations MA and LFC.

**Response:** We changed "LFC" to "Log fold change" and "MA plot" to "plot".

- lines 238-244. Since the previous study by this group quantified RNA expression in mixed, unsorted PBMCs, it is very difficult to compare those previous results with the present results in B cells, although it is noted that there was no great difference in the transcriptome of non-B cells in this study between the WT and ΔmiRNA-infected animals (which might therefore differ from the cow).

**Response:** We agree that it is very difficult to compare results from the bovine and ovine models. Comparison is hazardous because, in our previous study on the bovine species, B cell populations were not sorted prior to RNA sequencing. It is therefore possible that a series of significant genes in B-lymphocytes were not identified due to the transcriptome of non-B cells. This is discussed on page 12.

**Reviewer #2:**

- Fig 2C: are there common genes in the two groups? Or is the profile of non-B and B cells completely different?

**Response:** There are 11 common genes in the list of B and non-B cells. We now provide these data in Supplementary Table 1.

- Fig 2D: same comment as for 2C. Also, do the two viruses differentially downregulate host cell genes?

**Response:** The panel shows that 797 and 1361 genes are differentially down- and up-regulated by the two viruses.

- Line 283-284: It would be interesting to show the effect of miRNA depletion on different phases of the virus life cycle. The manuscript would benefit from these data.

**Response:** This would indeed be interesting. Unfortunately, available cell culture models are inadequate to analyze the virus life cycle (see also reply 3.2.).

**Reviewer #3:**

- the number of animals used for each experiment must be notified. Specially for fig 4-5-6 in which it is not clear how the stats were done.

**Response:** We have added the numbers in the figure legends.

**-** p19 lane 407-408 the sentence is not complete and seems to be a repetition of the previous one.

**Response:** Corrected.

**-** I suggest to show the data obtained for #1131 animal in which infection with miRNA deletant resulted to high PVL. How is the B-cell proliferation in this animal? Does the virus revert to wt ?

**Response:** As requested, we have added the BrdU kinetic data of sheep #1131 in Figure S7. The estimated proliferation rate is 0.08%. PCR of panel C shows that the virus did not revert to wild-type.
